# Supplementary material for: Metformin lotion promotes scarless skin tissue formation through AMPK activation, TGF-β1 inhibition, and reduced myofibroblast numbers
Source: PLoS One. 2024 Sep 27;19(9):e0311147. doi: 10.1371/journal.pone.0311147 (PMC11433050; doi:10.1371/journal.pone.0311147)
Supplement: Attachment — Submitted filename: Reviewer comment PONE-D-24-30549.docx [file pone.0311147.s001.docx]

19.08.2024

In this manuscript, the authors described "**Metformin Lotion Promotes Scarless Skin Tissue Formation through AMPK Activation, TGF-β1 Inhibition, and Reduced Myofibroblast Numbers**". The study will be beneficial for the literature. The report is an interesting study, but it needed some suggestions for publication.

**Here are the concerns for the authors;**

**Generally;**

1. The affiliations should be correctly written.
2. More information about metformin should be given in the introduction.
3. What is the purpose of choosing Metformin lotion? You should indicate in the article why you chose a lotion and not a cream, ointment or gel.
4. 6% ML was used according to what? Have any experiments been carried out to determine this concentration? If so, the results and explanations should be given.
5. It would be better if photographs could be added to be more descriptive about the wound study applied in rats.
6. “A 2 cm incised wound was carefully created on the skin over each Achilles tendon area using a scalpel, after which the wounds were sutured.”, no anesthesia was administered at this stage? It should be indicated how the anesthesia was administered and how much was given to rats.
7. G power analysis related to the animals used should be given.
8. Why was a healthy control group or a positive control group not included in the groups? Group 1 is a negative control in which a wound was created and given 0% ML. Therefore, would it not have been correct to compare it with a group without wound formation (healthy control) or with a lotion used routinely (positive control)?
9. “Achilles tendon area using a scalpel, after which the wounds were sutured.”, and what was done to prevent any contamination afterwards?
10. Fluorescent figures should be given sharper.
11. “Semi-quantification results indicate more than 46% of the cells around the wound area of the rat skin treated with 6% ML kept HMGB1 in the nuclei; however, less than 3.7% of the cells in the wound area of the rat skin treated with 0% ML showed HMGB1 in the nuclei”, figure legends contain bulleted sentences. These sentences should be included in the results section, not in the figure legends. Review the legends of each figure.
